# Slaughter Characteristics of Feedlot-Finished Premium South African Lamb: Effects of Sex and Breed Type

**DOI:** 10.3390/foods9050648

**Published:** 2020-05-18

**Authors:** Daniël André Van der Merwe, Tertius Swanepoel Brand, Louwrens Christiaan Hoffman

**Affiliations:** 1 Department of Animal Sciences, Stellenbosch University, Private Bag X1, Matieland 7602, South Africa; dvdmpok@gmail.com (D.A.V.d.M.); louwrens.hoffman@uq.edu.au (L.C.H.); 2 Directorate: Animal Sciences, Department of Agriculture, Western Cape Government, Private Bag X1, Elsenburg 7607, South Africa; 3 Centre for Nutrition and Food Sciences, Queensland Alliance for Agriculture and Food Innovation (QAAFI), The University of Queensland, Health and Food Sciences, Precinct, 39 Kessels Road, Coopers Plains 4108, Australia

**Keywords:** carcass composition, subcutaneous fat cover, maturity, fat-tailed breeds, meat quality

## Abstract

This study compared the carcass characteristics of ram and ewe lambs from South African wool, dual-purpose, meat, and fat-tailed sheep types, reared to produce premium lamb carcasses. The lambs were reared on a feedlot diet (10.41 MJ ME/kg feed and 19.06% crude protein) from weaning (30 kg live weight) until they attained a back-fat depth of ~4 mm, measured using ultrasound. After slaughter, the carcasses were assessed for retail cut yields, composition, and physical meat quality. Later, maturing lambs attained heavier carcass weights than early maturing breeds (~20.7 kg vs. 16.9 kg, respectively; *p* ≤ 0.05), and differences in carcass composition and retail cut yields were ascribed to differences in the frame size and pattern of fat deposition of the respective breeds. Small differences in physical meat quality were observed, with meat from Dormer and Namaqua lambs having notably higher shear-force values (~46 N) than that from Meatmaster carcasses (~34 N). These differences though are expected to have little influence on the perceived quality of the meat.

## 1. Introduction

Lamb consumers are generally concerned with the fattiness, tenderness, colour, and freshness of the purchased product, which is expected to be consistent [1]. In order to assist the consumer, carcass descriptors have been set by the South African carcass classification system [2] based on the age, fat cover, and conformation of the lamb carcass to provide information on the composition of the carcass as well as the expected quality characteristics with the fat cover being the main price determinant. Previous studies have shown how subcutaneous fat cover can be used as a predictor for carcass composition, particularly measurements taken on the *longissimus lumborum* muscle between the third and fourth lumbar vertebrae [3]. This specific measurement is used to classify carcasses in terms of tissue composition from no fat (fat score 0) to excessively fat (fat score 6). Prior to the institution of the classification, the industry relied on a grading system that defined the value of carcasses according to carcass standards [4,5]. The South African market has a high demand for lamb from young sheep (with no permanent incisors, Age Class A) and a lean fat cover (fat depth of 1–4 mm; Fat Class 2) [2], with about 72% of sheep slaughtered in registered abattoirs meeting these specifications [6]. While not being graded as premium lamb, there is a great demand for carcasses with these classifications (A2), and so a premium price is offered for carcasses that meet these specifications. Unlike the Australian and New Zealand systems which still acknowledge meat from sheep with two permanent incisors, that are not in wear, as lamb [7], the South African industry perceives lamb with no permanent incisors to be of premium quality, while yearling (two teeth) lambs still produce carcasses with acceptable eating quality characteristics [8].

In order to obtain a premium value for their carcasses, lamb producers make use of feedlot finishing soon after weaning in order to add value to their lambs in preparation for slaughter. From the viewpoint of a lamb producer, it is important to meet the market demands in order to enhance income from production and sustain optimum profitability. The South African sheep industry is made up of a number of breeds that are developed either for wool (Merino sheep) or meat (Dormer and Dorper breeds), as well as dual-purpose breeds (Dohne Merino and South African Mutton Merino) and fat-tailed sheep breeds (Meatmaster and Namaqua Afrikaner) which are able to survive and produce under more arid conditions. When considering that lambs of different breeds start to deposit fat at different stages as well as exhibit different growth capacities, it is important to implement precision finishing in maintaining production sustainability. Early maturing breeds deposit fat at an earlier stage and must, therefore, be slaughtered at a lighter live weight compared to later-maturing breeds for carcasses to obtain the same classification [9]. With the differences in slaughter weight, it is possible that the different breeds will not only differ in terms of carcass size and shape but could possibly differ in terms of meat quality characteristics, as it is not clear from previous studies [3,4] whether the effects of different breeds are accounted for by the quality descriptors. 

While the proposed slaughter weights of ~36 kg for Dorper lambs and 42–45 kg for dual-purpose and Merino lambs [9] have conventionally been used based on slaughter information, in an era of precision farming, technology can be used to give more accurate indications. Ultrasound scanning can be used to measure back-fat cover [10], and hence determine the optimal point of slaughter to achieve the desired classification in terms of subcutaneous fat cover. 

The aim of this study was to compare the carcass composition and physical meat quality characteristics of ewe and ram lambs from seven different South African sheep breed types, slaughtered at a recommended slaughter weight as determined by back-fat measurements using ultrasound scans to achieve the premium carcass classification (fat class 2; 1–4 mm subcutaneous fat measured at the 13th rib and between the third and fourth lumbar vertebrae). 

## 2. Materials and Methods

### 2.1. Animal Management

In this study, the carcass and physical meat quality characteristics of ewe and ram lambs from Dohne Merino, Dormer, Dorper, Meatmaster, Merino, Namaqua Afrikaner, and South African Mutton Merino (SAMM), slaughtered at a recommended slaughter weight (according to subcutaneous fat cover), were assessed. A total of 148 lambs were slaughtered, and carcasses evaluated, with the number of ewe and ram lamb carcasses for the respective breeds being presented in Table 1. The rearing and slaughter procedures followed in this study were approved by the Departmental Ethics Committee of Research on Animals (DECRA 14/110) of the Western Cape Department of Agriculture, South Africa.

The breeds were obtained from the same resource flock that was flocked together under the same conditions of the Western Cape Department of Agriculture’s Langgewens Research farm in the Swartland district of the Western Cape (coordinates: −33.276833, 18.704252). The ewes were synchronised and bred with rams from the respective breeds, so as to restrict the lambing period to within a month. After lambing, the flock with lambs was kept on cereal stubble with medics (*Medicago truncutula*, *Medicago*, *littoralis*, and *Medicago polymorpha*) pastures. In addition, lambs were allowed *ad libitum* access to creep feed (869.0 g/kg total digestible nutrients, 182.0 g/kg crude protein, 135.0 g/kg fat, 84.0 g/kg crude fibre, 11.2 g/kg calcium, and 7.4 g/kg total phosphorous) before weaning. The tails of Dohne Merino, Dormer, Dorper, Merino, and SAMM lambs were docked using a hot cauterising iron at two weeks of age, while that of Meatmaster and Namaqua lambs were left intact, as is commercial practice. The lambs were weighed on a weekly basis and weaned when they attained a live weight of 30 kg (27 kg for Namaqua Afrikaner lambs) when they were moved to be finished under feedlot conditions. In the feedlot, the lambs were reared on a concentrate feedlot diet (Table 2) *ad libitum*. Weekly weighing of the lambs continued with ultrasound (Mindray DP 30V, Shenzhen Mindray Bio-medical Electronics Co., Ltd.) scanning of the back-fat depth on the *Longissimus lumborum* muscle at the 13th rib and rump (between the third and fourth lumbar vertebrae).

### 2.2. Carcass Characteristics of A2 Lamb

The weekly scanning of back-fat depths of lambs was used in order to identify when the lambs were suitable for slaughter to obtain a carcass fat classification of 2 (~4 mm back-fat) (Government notice No. R. 863, 2006). Correction calibrations using the formula: US = 0.392F + 0.3631, R^2^ = 0.726 (where US is the ultrasound measurement, and F is the carcass fat depth in cm) [12] were used to predict carcass fat thickness from ultrasound scan measurements. From the above function, the recommended 4 mm carcass back-fat threshold equated to an ultrasound measurement of 5.2 mm. As lambs had to be identified for slaughter a week in advance, the threshold ultrasound back-fat measurement of 4.55 mm was used to identify lambs ready for slaughter. After identifying lambs ready for slaughter, they were kept in a feedlot for five days and on the sixth day, the lambs were weighed, scanned, and transported to a nearby (~40 km) registered commercial abattoir (Delico, Riebeek Kasteel, South Africa); these measurements were taken as the slaughter weights, and the final ultrasound scanned fat depths of the lambs. The lambs were transported in groups weekly, as they were identified to be ready for slaughter, by experienced transporters using a pickup truck and sheep trailer with a maximum load density of 20 lambs. At the abattoir, the lambs were held in lairage overnight for ~18 h before slaughter, with free access to water. The lambs were transported to the abattoir at the same time each week, by the same operator, and were kept in the lairage for the same period before being slaughtered the next morning, at the same time each week. As groups of lambs from the same farm were slaughtered at different times, as they were identified to be slaughter-ready, the transport and lairage conditions were kept relatively constant in order to allow comparisons of the carcasses at different times, with the same level of subcutaneous fat depth to be made.

At slaughter, the lambs were rendered unconscious by electrical stunning (JARVIS Electric Stunner; 200 V for 5 s) performed using stunning tongs applied to the head of the sheep (200 V for 5 s), before they were immediately exsanguinated and the carcasses were suspended at the hocks to assist bleeding out. No electrical stimulation was applied to the lambs at any point in the slaughter line. After bleeding out, the offal components (head, trotters, testes, skins, gastrointestinal tract, red offal, as well as abdominal fat) were removed. The warm carcass, containing kidneys and kidney fat, was then inspected and classed by a certified red meat classifier. The carcass classification was performed visually according to the description of the Government Notice No. R. 863 [2]. The pH and temperature of the left *longissimus lumborum* muscle were measured at the 13th rib 30 min post-mortem using a handheld pH meter (ACCSEN PH5 Food). The pH meter was calibrated using pH 4 and pH 7 buffer standards (ACCSEN) at room temperature (~24 °C for measuring the warm carcass and at 4 °C for cold carcass). The carcass temperature was recorded along with pH, and no temperature compensation was performed. To prevent rapid chilling, carcasses were gradually cooled in a cooling passage for 1–2 h post-slaughter before chilling at 2 °C for 24 h.

After 24 h, the lambs were transported in a refrigerated trailer at 4 °C to the laboratory. Upon arrival, the pH and temperature of the cold carcasses were once again measured as described. The cold carcasses were then weighed, and a block test was performed, dividing the carcasses into primal retail cuts. Cold carcass weight was expressed as a proportion of the slaughter weight to determine the dressing out percentage of the carcass. The kidneys and kidney fat were removed from the carcass prior to butchery into primal cuts, as described by previous butchery studies [13]. The primal cuts consisted of the neck, shoulders, ribs, loin, flanks, legs, and tails. The knee sections were sawn off 2.5 cm above the tip of the joint, from both the shoulders and legs and were regarded as the hocks. Trimmings consisted of non-aesthetic bloodied tissue and sections contaminated with gastrointestinal contents. The components were then weighed separately and expressed as a proportion yield of the carcass weight. The block tests were carried out by the same butcher using a knife to limit wastage; a handsaw was only used to remove the hocks. No trimming of excessive carcass fat was performed during the butchery of the lamb carcasses. 

A three-rib cut was made between the ninth and the 12th ribs on the left side of the animal, to include the ninth, 10th, and 11th ribs [14]. This cut extended from the spinal column up until the plane where the curvature of the ribs moves inward. This three-rib cut was weighed and dissected into bone, lean and fat tissue which were weighed separately and expressed as a proportion of the cut, so as to give an indication of the carcass composition. 

The left *Longissimus lumborum* muscle was excised from the loin cut and was used for physical quality measurements. The subcutaneous fat depth and muscle depth was measured at the positions of the 13th rib and between the third and fourth vertebrae using an electronic calliper. The fat and excess connective tissue were then trimmed from the muscle, and three 2.5 cm chops were cut from the muscle and allowed to bloom at 14 °C for 45 min. Surface colour measurements of the chops were taken using a digital calibrated handheld Colour-guide 45 °/0° colorimeter (aperture size 11 mm; illuminate/observer of D65/10°) (BYK-Gardner GmbH, Gerestried, Germany). The calibration of the colorimeter was done using the standards provided (BYK-Gardner), according to the manufacturer’s instructions. Three measurements were taken on the bloomed surface to determine the Commission Internationale de L’éclairage (CIE) colour space L* (lightness), a* (red-green range), and b* (blue-yellow range) values. The chroma (colour intensity) and hue angle (colour definition) values were calculated from the individual a* and b* values [15]. Cooking loss was determined by weighing two of the sample chops together, which were then inserted into a polyethylene bag. These samples were cooked in the bags, in a hot water bath at 80° C for 60 min. The bags were then removed from the water bath, the exuded water drained, and the samples were submerged in cold water to cool at 4 °C for an hour. The samples were then removed from the bags, blotted dry using paper towels, and weighed. The cooking loss was calculated as the difference in weight and expressed as a percentage of the original sample weight. The third chop was then used to determine drip loss by weighing the chop and suspending it from a wire in an inflated and sealed polyethylene bag, ensuring that the cut did not touch the sides of the bag. The bags were hung in a refrigerator at 4 °C for 24 h. The samples were then removed from the bags and blotted dry using paper towels to remove exudate moisture before weighing. The drip loss was then expressed as the percentage of weight lost over a 24 h period.

The Warner–Bratzler shear-force was determined on the cooked meat samples from the cooking loss analysis. For the shear-force analysis, five 2.5 cm cores (1.27 cm in diameter) were cut parallel to the meat fibres from the cooked meat samples. The shear-force of the core samples were determined using an Instron universal testing machine (Instron model 4444/H1028, Apollo Scientific cc, South Africa) fitted with a Warner–Bratzler attachment with a 1 mm thick triangular blade with a semi-circular cutting edge, which would cut the core sample perpendicular to the grain. The Instron machine was set to operate with a load cell of 2.000 kN at a speed of 200 mm/min. The shear-force values obtained were then expressed in Newton (N). The shear-force was taken as the average of the five cores analysed for each sample.

### 2.3. Statistical Analysis

Statistical analysis of the carcass quality data collected was performed using the PROC GLM of SAS Enterprise Guide version 7.1 [16]. The main effects of sex and breed were compared, as well as the interaction between these effects was tested. The differences between the effects were considered to be significant at the 95% confidence level (*p* ≤ 0.05) and tended to differ at the 90% confidence level (*p* ≤ 0.10). The calculations of the yields of the various components are described above. The type III sum of squares was used to analyse the data, with the various traits being expressed as least square means (LSM) with respective standard errors. The differences between effects were evaluated using the Bonferroni test at the 5% significance level.

## 3. Results

No significant interactions between the main effects of sex and breed were noted for the majority of the carcass and quality characteristics measured; therefore, only the main effects are presented in the tables with any interactions being described in the text. The slaughter ages of the ewes were older than that of the ram lambs (130 vs. 121 days, respectively; *p* = 0.008) (Table 3). Owing to their slower growth rates, Namaqua Afrikaner lambs were the oldest at slaughter (156 days), followed by Merino lambs (137 days), Dohne Merino lambs (126 days), which all differed from the Dormer lambs (110 days), which were the youngest at slaughter (*p* ≤ 0.05). With regard to the slaughter weight, an interaction (*p* ≤ 0.001) was observed between the effects of sex and breed which was primarily due to the ewe and ram lambs from the Namaqua Afrikaner breed not differing in slaughter weight (32.7 kg and 31.6 kg, respectively), while, ram lambs from the other breeds were generally heavier than the ewe lambs by a factor of ~11% (*p* ≤ 0.05) at the same scanned subcutaneous fat depth. A marked difference in slaughter weight between the breeds was observed, with the Dohne Merino, Dormer, Merino, and SAMM being heavier at slaughter (41.7–44.2 kg) and Namaqua Afrikaner lambs being the lightest (32.1 kg), with intermediate weights being observed for Dorper and Meatmaster lambs (37.9 and 35.1 kg, respectively). Ultrasound fat depth scans of the live lambs did not differ between ewes and rams at either of the scanning sites (*p* > 0.05). This was expected as a fat thickness of 5.2 mm at the rump region (third and fourth lumbar vertebrae) was taken as the indication for the lambs being ready for slaughter, with no differences observed for fat depth at this site (*p* > 0.05). However, at the position of the 13th rib, Merino lambs had the thickest fat depth (4.5 mm), which differed from that of the Dohne Merino (3.7 mm) and Namaqua Afrikaner lambs (3.2 mm) (*p* = 0.002). The cold carcass weights followed similar trends to that of the slaughter weight, including the interaction of Namaqua Afrikaner ewe and ram lambs not differing in carcass weight (16.0 and 14.2 kg, respectively; *p* > 0.05). Aside from the trends observed for the Namaqua lambs, generally, carcass weights of ram lambs were, on average, 7% heavier than that of the ewe lambs (*p* < 0.001). South African Mutton Merino lambs presented the heaviest carcasses, which differed considerably from Dorper carcass weights, which were, in turn, heavier than Meatmaster and Namaqua lamb carcasses (22.0 kg, 18.9 kg, 16.6 kg, and 15.1 kg, respectively). Carcass weights of Dohne Merino, Dormer, and Merino lambs did not differ from that of the SAMM lambs or the Dorper lambs (*p* > 0.05). The dressing percentage of ewe lambs was higher than that of ram lambs (49.1% vs. 47.2%, respectively; *p* < 0.001). The highest dressing percentage was obtained by the Dorper breed (49.9%) and the Dohne Merino and Namaqua breed presenting the lowest dressing percentages (46.9%; *p* ≤ 0.05). The fat class score of the different breeds differed (*p* ≤ 0.05), even while the animals were selected for slaughter to render a carcass with a fat score of 2. While the average fat score given to the carcasses across breeds was 2, the average score of the Meatmaster carcasses (2.6) was higher than that of the other breeds, apart from Namaqua carcasses (2.4), which only differed from that of Dohne Merino carcasses (1.9).

Traits relating to carcass composition were compared in Table 4. The fat and muscle tissue depths measured at both the 13th rib and the rump, using a calliper, did not differ between the sexes (*p* > 0.05), while the depth of these tissues did differ between breeds (*p* ≤ 0.05). The fat depth at the 13th rib was thickest for Merino lambs (3.97 mm), differing significantly from that of the Dohne Merino (2.54 mm) and Namaqua (2.29 mm) lambs. Similarly, the rump fat of Merino lambs was greater than that of Dormer lambs (7.54 mm vs. 5.78 mm, respectively). An interaction (*p* ≤ 0.05) was observed for muscle depth at the 13th rib, where the depths of Dormer, Dorper, and Namaqua ewes were 8.8%, 8.2%, and 26.7% greater than the ram lambs of the respective breeds, while the muscle depth of the sexes of the other breeds did not differ significantly. Muscle depth at the 13th rib of the Dormer and Dorper lambs (31.44 and 29.66 mm) was greater than that of Dohne Merino and Meatmaster lambs (26.72 and 25.34 mm), which, in turn, was greater than the muscle of the Namaqua lambs (20.82 mm). At the rump, the muscle depth of the Namaqua lambs (17.40 mm) was significantly thinner than that of the Dohne Merino, Dormer, and SAMM lambs (21.11–22.11 mm). At this site, the muscle depth of Dorper, Meatmaster, and Merino lambs did not differ from the other breeds (*p* > 0.05).

In terms of tissue composition estimated by the three-rib cut (Table 4), ewe lambs generally had a greater proportion of fat and lower proportion of lean and bone tissue (37.3%, 42.9%, and 19.4%, respectively) than that of ram lambs (33.1%, 44.8%, and 21.6%, respectively) (*p* ≤ 0.05). The Meatmaster lamb carcasses had the highest proportion of fat (39.8%), which differed considerably from that of the Dohne Merino and Dorper breeds (32.6% and 34.3%) and Namaqua lambs (29.2%) (*p* ≤ 0.05). The Dormer and Merino carcasses also had a greater proportion of fat (37.4%) than the Namaqua carcasses (*p* ≤ 0.05), while the proportion of fat of SAMM carcasses (35.5%) did not differ from that of the other breeds (*p* > 0.05). With regard to lean muscle tissue, Dorper and Namaqua carcasses presented a greater proportion of lean (46.6%) than Merino (41.5%) and Meatmaster (40.5%) carcasses, which had the lowest proportion of lean tissue (*p* ≤ 0.05). Dohne Merino and Namaqua Afrikaner carcasses consisted of the greatest proportion of bone tissue (22.0% and 23.6%), while the Dormer, Dorper, and Meatmaster carcasses had the lowest proportion of bone (18.4–19.4%). The proportion of bone in Merino and SAMM carcasses did not differ from that of the other breeds (*p* ≤ 0.05).

A butchery block test was performed to determine the yields of the primal retail cuts and waste trimmings from a lamb carcass (Table 5). Significant interactions between breed and sex were observed for the neck, tail, and kidney yields (*p* ≤ 0.05). The interaction in neck yields was as a result of the yields of the Dohne Merino and Dormer neck cuts not differing between the sexes (*p* > 0.05), while overall with the other breeds, ram carcasses presented larger neck yields than ewes (4.8% vs. 4.5%, respectively; *p* ≤ 0.05). The interaction in tail yield, was a result of differences (*p* ≤ 0.05) only being observed in breeds with intact tails (Namaqua Afrikaner and Meatmaster), whereas in breeds with docked tails, no differences were observed between ewes and rams (*p* > 0.05). As for the interaction in kidney yield, the ewe and ram carcasses from the Merino breed did not differ from each other (*p* > 0.05), whereas the ram carcasses from the other breeds had higher kidney yields than ewe carcasses. Overall, ram lamb carcasses had greater yields of neck, shoulders (16.7% vs. 16.2%), tail (1.9% vs. 1.6%), hocks (2.1% vs. 1.8%), and kidneys (0.7% vs. 0.6%), than ewe carcasses (*p* ≤ 0.05). Although Ewe carcasses presented higher yields of ribs (28.7% vs. 28.2%), and flank (6.7% vs. 6.2%) cuts than ram carcasses, the yields of loin and leg cuts, kidney fat, and trimmings did not vary significantly between the sexes.

Namaqua Afrikaner lamb carcasses presented the greatest (*p* ≤ 0.05) neck yields (5.4%), while the Dohne, Dormer, Dorper, and SAMM carcasses presented the lowest yields (4.3–4.4%). The shoulder cut yields were greatest in the Dohne Merino carcasses (17.4%), which did not differ markedly from that of the SAMM (17.1%) or Merino (16.7%) breeds. On the other hand, Dorper lambs had the lowest shoulder cut yields (15.7%), although they did not differ markedly from that of Namaqua, Dormer, and Meatmaster carcasses. Dormer carcasses presented the highest rib yields (29.4%), (*p* < 0.05) and Namaqua carcasses the lowest rib yields (26.2%). The highest loin cut yields were observed in the Dormer, Dorper, and Meatmaster carcasses (6.7–7.0%), which were greater (*p* ≤ 0.05) than that of the Dohne Merino, Merino, Namaqua, and SAMM carcasses (5.6–5.8%). The flank cut yields were highest in carcasses from the Meatmaster breed (7.2%), which differed from that of the Dohne Merino carcasses (6.2%), and the lowest yields were presented bythe Namaqua Afrikaner carcasses (5.3%; *p* ≤ 0.05). The highest yield of leg cuts was presented by the Dohne Merino and SAMM carcasses (32.1%), which differed from that of Merino (30.9%), Namaqua (30.2%), and Meatmaster carcasses (29.8%) with the lowest leg yield (*p* ≤ 0.05). The Namaqua Afrikaner carcasses had the greatest tail yields (6.0%) followed by the Meatmaster carcasses (2.1%), which were greater (*p* ≤ 0.05) than that of breeds with docked tails (0.7–1.0%), which did not differ from each other (*p* > 0.05). No significant differences were observed between breeds for hock yields, though a tendency (*p* = 0.053) was observed for the Dohne Merino and SAMM carcasses to have greater yields (~2.1%) and the Namaqua carcasses to exhibit lower hock yields (1.8%). Dorper carcasses had the lowest (*p* ≤ 0.05) kidney yields of the breeds (0.5%), which differed from that of the Dohne Merino, Meatmaster, Merino, and Namaqua Afrikaner breeds (0.7%). The kidney fat yield did not vary between the different breeds (*p* > 0.05). With regard to trimmings removed during butchery, the greatest yields were removed from the SAMM and Merino carcasses (0.5%) compared to the other breeds (0.4%; *p* ≤ 0.05).

The pH of the *longissimus lumborum* muscle measured 30 min post-mortem did not differ between the breeds (*p* > 0.05), although the muscle pH of ram lambs (6.84) was higher (*p* ≤ 0.05) than that of ewe lambs (6.69) (Table 6). An interaction was observed for the carcass temperature 30 min post-mortem (*p* = 0.012), where the temperature of SAMM ram carcasses was higher than ewe carcasses, while overall trends showed ewe carcasses were generally warmer (35.1 °C) than that of rams (33.7 °C; *p* ≤ 0.05). A tendency was observed (*p* = 0.086) for the temperature of SAMM carcasses to be warmer than Dohne Merino carcasses when measured 30 min post-mortem. When pH was measured 24 h post-mortem, no differences were observed between ram and ewe carcasses (*p* >0.05). However, a tendency (*p* = 0.051) was observed for Namaqua carcasses to have a higher pH 24 than the Dormer, Meatmaster, and SAMM carcasses (pH of 5.74, 5.52, 5.56, and 5.54, respectively). The temperature measured 24 h post-mortem also did not differ between sexes (*p* > 0.05), while the temperature of the Namaqua carcasses (6.1 °C) was significantly higher than that of the Dorper carcasses (4.4 °C). While cooking loss of muscles from ram carcasses was higher than that of ewes (39.6% vs. 38.4%, respectively; *p* ≤ 0.05), an interaction was observed (*p* = 0.024), where the cooking losses of the Dohne Merino and SAMM rams did not differ from that of the ewes. Muscles from the Dormer carcasses presented higher cooking losses than that from Merino and Namaqua (40.2% vs. 38.3%, respectively; *p* ≤ 0.05); cooking losses from the remaining breeds did not differ from each other (*p* > 0.05). The effects of sex and breed did not influence (*p* > 0.05) drip loss (1.1–1.4%). Also, sex did not influence the shear-force values (*p* > 0.05), while the shear-force of the Dormer (46.56 N) and Namaqua (46.09 N) samples was markedly higher than that of the Meatmaster (34.89 N). The shear-force values of the remaining breeds did not differ from the other groups (*p* > 0.05). 

The meat colour attributes of the *Longissimus lumborum* samples from the various lamb breeds are depicted in Table 7. The only attribute that was affected by sex was the lightness parameter L*, where ram lambs presented higher L* values than ewes (38.46 vs. 37.26, respectively; *p* < 0.001). Samples from the Namaqua and Dorper lambs were significantly darker than that of Dormer, Meatmaster, and SAMM breeds (36.28 and 36.85 vs. 38.85, respectively). The highest redness values (a*) were recorded for the Namaqua and Dorper samples (14.15 and13.82, respectively) and the significantly lowest for the Dormer and SAMM samples (12.64 and 12.59, respectively). The redness values of the Dohne Merino, Meatmaster, Merino, and SAMM breeds did not differ from any of the breeds (*p* > 0.05). No significant differences were observed between the samples of the different breeds for the yellowness parameter (b*). The hue angle of samples from the SAMM lambs (40.96) was markedly higher than that of the Dorper and Namaqua lambs (36.54° and 36.38°, respectively), while the values of the other breeds did not differ from any of the other breeds (*p* > 0.05). The chroma values of Namaqua lamb samples were higher than that of SAMM and Dormer samples (17.60, 16.73 and 16.44, respectively; *p* ≤ 0.05). 

## 4. Discussion

The South African red meat classification system has set standards to give consumers a description of the quality characteristics that can be associated with lamb carcasses given a specific classification [4]. The system for classing lamb carcasses is based on age (according to dentition) and the subcutaneous fat depth between the third and fourth lumbar vertebrae; though, it does not account for differences between breeds. Breeds developed from indigenous fat-tailed or fat-rump breeds tend to be early maturing and tend to deposit fat at an earlier age, as is the case with the Dorper, Meatmaster, and Namaqua breeds. These breeds also show differences in fat partitioning between the various fat depots, as well as the distribution of subcutaneous fat [9,17]. Breeds also vary in their growth potential in terms of frame size and growth rate. 

As the lambs in this study were reared under optimal growth conditions, they were ready for slaughter at a young age (soon after weaning at 100 days of age). Namaqua Afrikaner lambs that have a relatively lower mature weight [18] and so exhibit slower growth rates were only slaughter-ready after 150 days of age. As expected, the slaughter weights of early maturing breeds were lighter than the later maturing breeds, to obtain a carcass with the recommended fat coverage (Table 3). Ewe lambs were slaughtered at lighter body weights than ram lambs, due to ewes maturing earlier than rams, and depositing fat at a lower body weight [19]. In this study, contradicting results were observed with the slaughter weights of Namaqua Afrikaner ewes being slightly heavier, while not differing significantly compared to that of the ram lambs. In fat-tailed sheep, fat is primarily deposited in the tail depot, as the name states before subcutaneous fat is deposited on the rump and the rest of the body [17]. Therefore, the back-fat measurement sites used in this study may not give an apt indication of the level of maturity, in terms of fatness, for this breed with low mature weights, which is early maturing but deposits the majority of fat in other depots. Further research may be needed to describe the differences in body fat deposition of Namaqua Afrikaner sheep of different sexes. Overall, the differences in slaughter weight were carried through to the cold carcass weights of the respective breeds even while the dressing percentages of the different breeds varied. During slaughter, the additional weight of the removed testes from rams contributed to the offal component and resulted in rams having lower dressing percentages than ewes. The Dorper lambs exhibited the highest carcass dressing percentages as a result of higher levels of fat cover throughout the carcass, while the Dohne Merino and Namaqua Afrikaner carcasses had leaner (*p* ≤ 0.05) fat depths proximal to the 13th rib (Table 5). The dressing percentage of the carcass increases with the level of subcutaneous fat cover, and so lambs with a greater distribution of subcutaneous fat will present a higher yield [9]. The tissue composition of the carcasses from the different groups varied. The Meatmaster and Namaqua carcasses were given higher average subjective fat scores due to the presence of the fat tail, which might have caught the attention of the classifiers as they viewed the hanging carcasses, even while the back-fat scans at the rump regions did not differ between the breeds prior to slaughter. While the red meat carcass classification system refers to the fat depth at the rump measuring site [2], which correlates with the carcass tissue composition [3]; carcass classifiers consider the carcass as a whole, looking at the fat cover over the ribs and brisket, loin and rump as well as tail regions. Therefore, due to the abundance of fat associated with the tail of the fat-tailed breeds, classifiers do tend to class these carcasses with a higher fat score.

Both measurements from the scans prior to the slaughter and the measurements taken of the muscle after chilling showed that the fat depths differed at the two measurement sites, with the carcasses tending to become leaner as one moves proximal from the rump region. At the same time, the muscle (*Longissimus lumborum*) depths increased as one moved proximal from the rump towards the 13th rib. Cloete et al. [20] also observed that the fat depth measured at the 13th rib was not as thick as that at the rump for the Dohne Merino, Merino, Dormer, and SAMM sheep. Allometric coefficients for subcutaneous fat in European breeds were highest on the rib-loin regions, followed by the breast and chump, with lower coefficients being associated with the leg, shoulder, and neck cuts [21]. However, this study shows that greater fat depths are observed nearer to the rump while at the ribs, thinner subcutaneous fat depths were observed. Muscle depths did not exhibit any differences between sexes; the muscle depths of meat-type breeds with improved conformation (Dormer, Dorper and SAMM) were greater hinting towards improved musculature of the carcasses. According to the three-rib cut used to estimate tissue composition, carcasses from ram lambs were leaner and had higher proportions of lean and bone tissue than that of ewe lambs. This can again be attributed to the fact that female animals attain maturity earlier than male counterparts, and thus, are overall fatter when slaughtered at the same stage [22,23]. Interestingly, the lighter Namaqua Afrikaner carcasses were the leanest and had the greatest proportions of lean and bone tissue. This is due to the majority of the fat of the breed being deposited in the tail and rump depots [17], while the fat distribution of the carcass becomes leaner as one moves proximally from the rump. From the three-rib cut, the Dorpers also appeared relatively leaner, with greater muscle yield and more favourable muscle to bone ratio. Dohne Merino carcasses were found to have similar fat and lean muscle yields to that of Dorper; however, they did present a less favourable muscle to bone ratio, as did Merino and SAMM carcasses. The Meatmaster carcasses had higher proportions of fat and lower proportions of lean muscle, which relates to the lower muscle depths measured in carcasses from this breed. Dormer carcasses were found to have fat and muscle yields resembling the higher levels observed in this study, while also having a lower bone tissue yield. Evaluating the ratio of lean:fat in the saleable meat portion predicted by the three-rib cut, Namaqua carcasses are relatively leaner (1.59:1) followed by Dohne Merino and Dorper carcasses (~1.36:1), SAMM and Dormer carcasses (~1.19:1), while Merino (1.11:1) and Meatmaster (1.02:1) carcasses presented a higher degree of fatness relative to lean meat yield. In terms of carcass conformation, by combining the yields of fat and lean meat tissue and expressing it relative to that of bone, Dormer lambs have the greater conformation (4.4:1) followed by Dorper (4.3:1), Meatmaster (4.1:1), SAMM and Merino (3.8:1), Dohne Merino (3.5:1) and Namaqua lambs with the lowest relative conformation (3.2:1). It should be considered that conformation expressed in this manner is relative to the yields of the carcass tissues from the three-rib cut and does not take the size of the carcass into account, which also contributes to the conformation. Under the South African carcass classification system, the conformation score is viewed as a descriptor but does not carry importance to the financial value of the carcass as is the case with fat cover and carcass weight as it is argued that conformation will be reflected in the carcass weight; while the yield of the retail cuts determines the overall value of lamb meat production.

Lamb is considered to be a high-value red meat commodity with cuts from the leg and loin, usually contributing the highest values to the carcass. Aside from the leg and loin cuts, the rib and shoulder cuts are deemed to be higher value cuts, whereas stewing meat associated with the neck, flank, and shin are typically regarded as lower value lamb cuts. Due to the popularity of lamb tails as a novelty barbecue snack and for the use of tail-fat in the making of beef and game droëwors [24], the relative value of this once off-cut has now increased. The traditional butchers’ block test is used to determine the yields of specific cuts (primal or secondary cuts), and then by incorporating market values to the cuts, the profitability of carcass butchery can be determined. Due to the differences in carcass weight and frame size, the relative primal retail cut yields of the carcasses from different breeds with the same fat score differed. The most notable differences are the tail cut yields of the Meatmaster and Namaqua Afrikaner carcasses compared to the other breeds. This is firstly due to the tails of the other breeds being docked within two weeks post-partum, and secondly, the enhanced fat deposition in the tail depot. The tail fat on average contributes 28.8% of total dissectible fat in fat-tailed breeds [17]. Ewe lambs were also observed to have smaller tails than that of rams; this may possibly be a physiological adaptation of the breeds to assist rams in tail-lifting during mating for improved reproductive success [25]. Owing to their smaller frame size and lack of conformation, Namaqua Afrikaner carcasses had low rib and shoulder yields. These cuts in Namaqua lambs also have a higher percentage of bone and less meat than Dorper or SAMM carcasses [26]. The lower shoulder yield in Dorper carcasses may be as a result of the shorter wither height of the breed in relation to other breeds of the same frame size. However, leg length was not measured in this study to confirm this. Breeds with better body conformation (SAMM, Dohne Merino, Dormer, and Dorper) presented higher leg cut yields due to improved muscling, while Dorper, Dormer, and Meatmaster lambs had the highest loin cut yields relative to carcass weight.

While carcass muscle pH after slaughter and after chilling did not differ between the breeds, differences in CIE Lab surface colour parameters were observed. The muscle surface colour of samples from the Dormer, Meatmaster, and SAMM lambs was lighter and had lower redness (a* values) with generally higher hue angles and lower chroma values. Meat from the Namaqua Afrikaner and Dorper lambs was generally darker with greater redness values, lower hue angles, and greater chroma saturation values (Table 7). With regard to the effect of sex, meat from ram lambs was lighter than that of ewes. Cloete et al. [20] also observed meat from Dormer sheep to be lighter than that of the Merino, Dohne Merino, and SAMM sheep, while the other colour parameter values did not differ. On average, Australian consumers accepted lamb meat with an L* value of 34 and a* value of 9.5 while 95% of consumers still find lamb meat with an L* = 44 and a* = 14.4 still acceptable [27]. The colour parameter values of meat from lamb breeds in this study were higher than the average threshold values reported by Khliji et al. [27] and so consumers would consider the colour of the lamb meat to be acceptable.

As muscle pH did not differ, drip loss also did not differ between the breeds. Although, meat sampled from Dormers had a significantly higher cooking loss than that of the Merino and Namaqua Afrikaner lambs. As a result of the greater cooking loss in Dormer samples, higher shear-force values were also observed. This is partly due to the density of muscle fibres increasing within the cooked meat sample as moisture is lost. Though, Hoffman et al. [28] reported that Warner–Bratzler shear-force of meat from Dormer crossbreed lambs was greater than that of crosses with other breeds, and so suggesting a possible breed effect. Shear-force values from Namaqua lambs resembled that of the Dormers. These shear-force values, though do not correlate with tough meat but rather fall into the intermediate category (42.87–52.68 N), while samples from the other breeds can be regarded as tender (32.96–42.77 N) [29].

The role of carcass classification is to set descriptors so that carcasses are classified to ensure more consistent meat quality [5]. Consumers desire a meat product that is lean but still contains sufficient fat to ensure a good eating experience [30]. Therefore, the South African market is driven to produce a lamb with a carcass fat classification of 2, which also ensures minimal trimming of fatter carcasses and so reducing profitability [31]. While the aim is to produce a lamb carcass with uniform quality and composition, the fat cover does vary across the carcass [31] with patterns of fat deposition in the carcass and non-carcass depots varying between breeds, particularly in fat-tailed breeds [17,26,32]. The frame size or mature weights of the various breeds differ [33], and so body conformation and musculature will vary, resulting in differences in tissue composition as well as the yield of carcass cuts. Under the same rearing conditions, objectively measured differences in physical meat quality characteristics were observed between breeds. However, the range of variability observed for these traits still falls within an acceptable range for consumers. The impact of producing lamb from different breeds would have a greater effect on the profitability of meat processors than on end consumers. Certain markets though have preference for lamb carcasses of different sizes, depending on the products they promote. Thus, there is place in the value chain for each of the sheep breed types for lamb production with strategic marketing. Using the results of this study as a guideline, taking into account available resources and implementing specific strategies, sustainable feedlot lamb production can be accomplished, with processors also having to strategize their carcass butchery for optimal profitability.

## 5. Conclusions

Differences in maturity and the onset of fat deposition account for the differences in carcass weights for the different breeds. Although the carcass classification system is well suited to describe the carcass of most breeds, it must be considered that with regard to fat-tailed breeds, while proximal to the rump, the carcasses are relatively lean, the fat deposition surrounding the tail and rump does influence the decision of the carcass classifiers. The different breeds exhibit different frame sizes and body conformations at the recommended degree of carcass fat cover. This influences the yields of specific cuts and the degree of lean muscle within the cuts.

Within the same carcass fat class, small differences in physical meat quality characteristics instrumental shear-force were observed, though, these differences are not expected to compromise the perceived quality of the meat from different breeds. Therefore, slaughtering lambs of different breeds, at the same degree of fatness, results in lambs being reared for different periods, to obtain a carcass that varies in size and conformation; though still presenting similar meat quality characteristics.

## Figures and Tables

**Table 1:** Numbers of ewe and ram lamb carcasses assessed for the South African sheep breeds slaughtered at an ideal slaughter weight to produce premium lamb carcass.

Sheep Breed	Ewe	Ram	Total
Dohne Merino	19	14	33
Dormer	8	11	19
Dorper	11	16	27
Meatmaster	14	16	30
Merino	6	8	14
Namaqua Afrikaner	7	6	13
South African Mutton Merino	4	8	12
Total	69	79	148

**Table 2:** Ingredient formulation and nutritional composition of feedlot diet fed to lambs during the study period.

**Ingredient**	**Inclusion (g/kg as fed)**
Maize	500.0
Lucerne hay	361.0
Cottonseed oilcake	50.0
Molasses powder	25.0
Ammonium chloride	5.0
Ammonium sulphate	5.0
Lime	5.0
Monocalcium phosphate	5.0
Common salt	10.0
Urea	5.0
Sodium Bicarbonate	10.0
Slaked lime	5.0
Sulphur	2.0
Vitamin and mineral premix	1.5
Commercial growth promoters and coccidiostat premix *	1.2
**Nutrient**	**Contents**
Dry matter, g/kg	901.5
Total digestible nutrients (TDN) ^1^, g/kg	694.3
Metabolisable energy, MJ/kg	10.41
Nitrogen free extract ^2^, g/kg	393.6
Crude protein, g/kg	190.6
Rumen undegradable protein (RUP) ^3^, g/kg	43.0
Crude fibre, g/kg	152.2
Neutral detergent fibre, g/kg	237.9
Acid detergent fibre, g/kg	170.2
Ash, g/kg	102.3
Fat, g/kg	62.6
Calcium, g/kg	13.9
Phosphorous, g/kg	4.3

* Premix contains Stafac, Selinomycin, and Taurotec. ^1^ Calculated total digestible nutrients = (0.8 protein) + (0.4 fibre) + (0.9 nitrogen free extract) + (2.025 fat). ^2^ Calculated Nitrogen free extract = 100–(moisture +ash + protein + crude fibre + fat). ^3^ RUP calculated from protein degradability values for maize (63.0%), lucerne meal (68.9%) and cottonseed oilcake (54.5%), at an outflow rate of 0.05/hr [11].

**Table 3:** Slaughter characteristics of A2 carcasses depending on the lamb sex and breed at a fat scan thickness of 5.2 mm, expressed as the least square means ± standard error.

Main Effect		Slaughter Age (days)	Slaughter Weight (kg)	Fat Scan 13th rib (mm)	Fat Scan Rump (mm)	Cold Carcass Weight (kg)	Dressing Percentage (%)	Fat Class *
Sex	Ewe	130 ± 2.3	37.5 ± 0.48	3.9 ± 0.10	5.0 ± 0.08	18.4 ± 0.24	49.1 ± 0.31	2.2 ± 0.05
	Ram	121 ± 1.9	41.7 ± 0.43	3.8 ± 0.09	5.0 ± 0.07	19.7 ± 0.22	47.2 ± 0.28	2.2 ± 0.04
	*p*-value	0.008	<0.001	0.625	0.510	<0.001	<0.001	0.466
Breed	Dohne Merino	126 ^bc^ ± 2.7	43.7 ^a^ ± 0.63	3.7 ^bc^ ± 0.13	4.9 ± 0.10	20.4 ^ab^ ± 0.31	46.9 ^c^ ± 0.40	1.9 ^c^ ± 0.07
	Dormer	110 ^d^ ± 3.6	42.7 ^a^ ± 0.83	4.0 ^abc^ ± 0.18	4.8 ± 0.13	20.5 ^ab^ ± 0.42	48.0 ^abc^ ± 0.53	2.1 ^bc^ ± 0.09
	Dorper	115 ^cd^ ± 3.1	37.9 ^b^ ± 0.70	4.0 ^abc^ ± 0.15	5.2 ± 0.11	18.9 ^b^ ± 0.35	49.9 ^a^ ± 0.45	2.1 ^bc^ ± 0.07
	Meatmaster	120 ^cd^ ± 2.9	35.1 ^bc^ ± 0.67	3.7 ^abc^ ± 0.15	5.3 ± 0.11	16.6^c^ ± 0.34	47.4 ^bc^ ± 0.43	2.6 ^a^ ± 0.07
	Merino	137 ^ab^ ± 4.2	41.7 ^a^ ± 0.96	4.5 ^a^ ± 0.20	4.9 ± 0.15	20.0 ^ab^ ± 0.48	48.0 ^abc^ ± 0.62	2.2 ^bc^ ± 0.10
	Namaqua Afrikaner	156 ^a^ ± 5.7	32.1 ^c^ ± 0.99	3.2 ^c^ ± 0.21	5.1 ± 0.16	15.1 ^c^ ± 0.50	46.9 ^c^ ± 0.64	2.4 ^ab^ ± 0.10
	South African Mutton Merino	114 ^cd^ ± 4.8	44.2 ^a^ ± 1.09	3.8 ^abc^ ± 0.23	4.8 ± 0.17	22.0 ^a^ ± 0.55	49.8 ^ab^ ± 0.70	2.0 ^bc^ ± 0.11
	*p*-value	<0.001	<0.001	0.002	0.078	<0.001	<0.001	<0.001
S × B interaction	*p*-value	0.609	<0.001	0.518	0.085	<0.001	0.339	0.850

^a–d^ Column means with different superscripts differ significantly (*p* ≤ 0.05) * Average fat classification according to subcutaneous fat depth cover, Class 0 (no fat), Class 1 (<1 mm), Class 2 (1–4 mm), Class 3 (4–7 mm), Class 4 (7–9 mm), Class 5 (9–11 mm) and Class 6 (>11 mm) (Government notice No. R. 863, 2006).

**Table 4:** Fat and muscle depth and the three-rib tissue composition of A2 carcasses depending on the lamb sex and breed, expressed as the least square means ± standard error.

Main Effect		Rib Fat Depth (mm)	Rump Fat Depth (mm)	Rib Muscle Depth (mm)	Rump Muscle Depth (mm)	Fat Tissue (%)	Lean Tissue (%)	Bone Tissue (%)
Sex	Ewe	3.26 ± 0.136	6.59 ± 0.209	27.04 ± 0.455	20.21 ± 0.441	37.3± 0.75	42.9± 0.58	19.4± 0.40
	Ram	2.96 ± 0.121	6.58 ± 0.186	26.31 ± 0.405	20.01 ± 0.392	33.1± 0.67	44.8± 0.52	21.6± 0.35
	*p*-value	0.098	0.958	0.231	0.723	<0.001	0.011	<0.001
Breed	Dohne Merino	2.54 ^b^ ± 0.177	6.08 ^ab^ ± 0.271	26.72 ^b^ ± 0.592	21.71 ^a^ ± 0.573	32.6 ^bc^ ± 0.97	44.8 ^ab^ ± 0.75	22.0 ^a^ ± 0.52
	Dormer	3.37 ^ab^ ± 0.233	5.78 ^b^ ± 0.358	31.44 ^a^ ± 0.781	21.11 ^a^ ± 0.756	37.4 ^ab^ ± 1.28	43.9 ^abc^ ± 0.99	18.4 ^b^ ± 0.68
	Dorper	3.11 ^ab^ ± 0.196	6.12 ^ab^ ± 0.302	29.66 ^a^ ± 0.658	18.54 ^ab^ ± 0.637	34.3 ^bc^ ± 1.08	46.6 ^a^ ± 0.84	18.6 ^b^ ± 0.57
	Meatmaster	3.27 ^ab^ ± 0.189	6.86 ^ab^ ± 0.291	25.34 ^b^ ± 0.635	18.93 ^ab^ ± 0.615	39.8 ^a^ ± 1.04	40.5 ^c^ ± 0.81	19.4 ^b^ ± 0.55
	Merino	3.97 ^a^ ± 0.271	7.54 ^a^ ± 0.416	24.50 ^bc^ ± 0.907	20.99 ^ab^ ± 0.878	37.3 ^ab^ ± 1.49	41.5 ^bc^ ± 1.16	20.7 ^ab^ ± 0.79
	Namaqua Afrikaner	2.29 ^b^ ± 0.279	6.66 ^ab^ ± 0.429	20.82 ^c^ ± 0.935	17.40 ^b^ ± 0.905	29.2 ^c^ ± 1.53	46.6 ^a^ ± 1.19	23.6 ^a^ ± 0.82
	South African Mutton Merino	3.24 ^ab^ ± 0.307	7.07 ^ab^ ± 0.472	28.27 ^ab^ ± 1.029	22.11 ^a^ ± 0.996	35.5 ^abc^ ± 1.69	43.0 ^abc^ ± 1.31	20.8 ^ab^ ± 0.90
	*p*-value	<0.001	0.011	<0.001	<0.001	<0.001	<0.001	<0.001
S × B interaction	*p*-value	0.817	0.272	0.026	0.054	0.283	0.36	0.260

^a–c^ Column means with different superscripts differ significantly (*p* ≤ 0.05).

**Table 5:** Primal retail cut yields (as a proportion of cold carcass weight) of A2 carcasses, depending on the effect of lamb sex and breed, expressed as the least square means ± standard error.

Main Effect		Neck (%)	Shoulders (%)	Ribs (%)	Loin (%)	Flanks (%)	Legs (%)	Tail (%)	Hocks (%)	Kidneys (%)	Kidney Fat (%)	Trimmings (%)
Sex	Ewe	4.5 ± 0.05	16.2 ± 0.12	28.7 ± 0.15	6.3 ± 0.11	6.7 ± 0.10	31.1 ± 0.15	1.6 ± 0.08	1.8 ± 0.03	0.6 ± 0.01	2.0 ± 0.22	0.4 ± 0.02
	Ram	4.8 ± 0.05	16.7 ± 0.11	28.2 ± 0.13	6.2 ± 0.09	6.2 ± 0.09	31.2 ± 0.13	1.9 ± 0.07	2.1 ± 0.03	0.7 ± 0.01	1.7 ± 0.20	0.4± 0.02
	*p*-value	<0.001	0.002	0.011	0.312	<0.001	0.836	0.003	<0.001	<0.001	0.300	0.088
Breed	Dohne Merino	4.4 ^c^ ± 0.07	17.4 ^a^ ± 0.16	28.6 ^ab^ ± 0.19	5.7 ^b^ ± 0.14	6.2 ^c^ ± 0.13	32.1 ^a^ ± 0.20	0.7 ^c^ ± 0.10	2.0 ± 0.04	0.7 ^ab^ ± 0.02	1.9 ± 0.29	0.4 ^b^ ± 0.02
	Dormer	4.3 ^c^ ± 0.09	16.4 ^bcd^ ± 0.20	29.4 ^a^ ± 0.25	6.9 ^a^ ± 0.18	6.6 ^abc^ ± 0.17	31.1 ^abc^ ± 0.26	0.8 ^c^ ± 0.13	1.9 ± 0.05	0.6 ^bc^ ± 0.02	1.3± 0.39	0.4 ^b^± 0.03
	Dorper	4.4 ^c^ ± 0.08	15.7 ^d^ ± 0.17	28.3 ^b^ ± 0.21	7.0 ^a^ ± 0.15	6.9 ^ab^ ± 0.14	31.8 ^ab^ ± 0.22	1.0 ^c^ ± 0.11	1.9 ± 0.04	0.5 ^c^ ± 0.02	1.9± 0.33	0.4 ^b^± 0.03
	Meatmaster	4.8 ^b^ ± 0.07	16.2 ^bcd^ ± 0.17	28.6 ^ab^ ± 0.20	6.7 ^a^ ± 0.15	7.2 ^a^ ± 0.13	29.8 ^d^ ± 0.21	2.1 ^b^ ± 0.11	1.9 ± 0.04	0.7 ^ab^ ± 0.02	2.5± 0.31	0.4 ^b^± 0.03
	Merino	4.9 ^b^ ± 0.10	16.7 ^abc^ ± 0.24	29.0 ^ab^ ± 0.29	5.6 ^b^ ± 0.21	6.6 ^abc^ ± 0.19	30.9 ^bc^ ± 0.30	0.8 ^c^ ± 0.15	1.9 ± 0.06	0.7 ^a^ ± 0.03	2.5± 0.45	0.5 ^a^± 0.04
	Namaqua Afrikaner	5.4 ^a^ ± 0.11	15.8 ^cd^ ± 0.25	26.2 ^c^ ± 0.30	5.8 ^b^ ± 0.22	5.3 ^d^ ± 0.20	30.2 ^cd^ ± 0.31	6.0 ^a^ ± 0.16	1.8 ± 0.06	0.7 ^ab^ ± 0.03	1.3± 0.46	0.4 ^b^± 0.04
	South African Mutton Merino	4.3 ^c^ ± 0.12	17.1 ^ab^ ± 0.27	28.8 ^ab^ ± 0.33	5.8 ^b^ ± 0.24	6.4 ^bc^ ± 0.22	32.1 ^ab^ ± 0.34	0.7 ^c^ ± 0.17	2.1 ± 0.06	0.6 ^bc^ ± 0.03	1.6± 0.51	0.5 ^a^± 0.04
	*p*-value	<0.001	<0.001	<0.001	<0.001	<0.001	<0.001	<0.001	0.053	<0.001	0.156	0.036
S × B interaction	*p*-value	0.011	0.705	0.042	0.107	0.492	0.378	<0.001	0.090	0.035	0.850	0.069

^a–d^ Column means with different superscripts differ significantly (*p* ≤ 0.05).

**Table 6:** Physical meat quality characteristics of the *Longissimus lumborum* muscles excised from A2 carcasses depending on the effect of lamb sex and breed expressed as the least square means ± standard error.

Main Effect		pH 30	Temperature 30 (°C)	pH 24	Temperature 24 (°C)	Cook Loss (%)	Drip Loss (%)	Shear-Force (N)
Sex	Ewe	6.69 ± 0.046	35.1 ± 0.38	5.57 ± 0.025	5.0 ± 0.19	38.4 ± 0.24	1.3 ± 0.05	41.27 ± 1.28
	Ram	6.84 ± 0.040	33.7 ± 0.34	5.64 ± 0.023	5.2 ± 0.17	39.6 ± 0.21	1.2 ± 0.05	39.60 ± 1.14
	*p*-value	0.017	0.006	0.167	0.529	<0.001	0.206	0.645
Breed	Dohne Merino	6.86 ± 0.060	33.1 ± 0.50	5.64 ± 0.033	5.5 ^ab^ ± 0.24	38.6 ^ab^ ± 0.31	1.4 ± 0.07	38.92 ^ab^ ± 1.66
	Dormer	6.88 ± 0.079	34.8 ± 0.66	5.52 ± 0.044	4.7 ^ab^ ± 0.32	40.2 ^a^ ± 0.40	1.3 ± 0.09	46.56 ^a^ ± 2.19
	Dorper	6.65 ± 0.066	34.6 ± 0.55	5.59 ± 0.037	4.4 ^b^ ± 0.27	39.8 ^ab^ ± 0.34	1.3 ± 0.07	39.64 ^ab^ ± 1.85
	Meatmaster	6.69 ± 0.064	35.1 ± 0.53	5.56 ± 0.035	5.3 ^ab^ ± 0.26	39.5 ^ab^ ± 0.33	1.4 ± 0.07	34.89 ^b^ ± 1.78
	Merino	6.87 ± 0.091	34.0 ± 0.76	5.65 ± 0.051	5.3 ^ab^ ± 0.37	38.3 ^b^ ± 0.47	1.4 ± 0.10	36.62 ^ab^ ± 2.55
	Namaqua Afrikaner	6.75 ± 0.094	33.8 ± 0.79	5.73 ± 0.052	6.1 ^a^ ± 0.38	38.3 ^b^ ± 0.48	1.2 ± 0.11	46.09 ^a^ ± 2.62
	South African Mutton Merino	6.68 ± 0.103	35.3 ± 0.86	5.54 ± 0.057	4.6 ^ab^ ± 0.42	38.6 ^ab^ ± 0.53	1.1 ± 0.12	40.34 ^ab^ ± 2.89
	*p*-value	0.102	0.086	0.051	0.004	0.002	0.206	<0.001
S × B interaction	*p*-value	0.817	0.011	0.089	0.348	0.024	0.653	0.329

^a,b^ Column means with different superscripts differ significantly (*p* ≤ 0.05).

**Table 7:** Meat colour characteristics of *longissimus lumborum* muscles excised from A2 carcasses depending on the effects of lamb sex and breed expressed as the least square means ± standard error.

Main Effect		L*	a*	b*	Hue (°)	Chroma
Sex	Ewe	37.26 ± 0.239	13.36 ± 0.143	10.47 ± 0.144	38.18 ± 0.474	17.02 ± 0.147
	Ram	38.46 ± 0.213	13.41 ± 0.128	10.52 ± 0.128	38.12 ± 0.422	17.08 ± 0.131
	*p*-value	<0.001	0.796	0.804	0.916	0.743
Breed	Dohne Merino	38.12 ^ab^ ± 0.311	13.47 ^ab^ ± 0.186	10.30 ± 0.187	37.38 ^ab^ ± 0.617	16.97 ^abc^ ± 0.191
	Dormer	38.54 ^a^ ± 0.410	12.64 ^b^ ± 0.247	10.46 ± 0.247	39.63 ^ab^ ± 0.814	16.44 ^c^ ± 0.252
	Dorper	36.85 ^bc^ ± 0.346	13.82 ^a^ ± 0.207	10.23 ± 0.208	36.54 ^b^ ± 0.686	17.23 ^ab^ ± 0.213
	Meatmaster	38.84 ^a^ ± 0.334	13.67 ^ab^ ± 0.200	10.74 ± 0.201	38.20 ^ab^ ± 0.662	17.41 ^ab^ ± 0.205
	Merino	37.54 ^abc^ ± 0.477	13.35 ^ab^ ± 0.285	10.44 ± 0.287	37.97 ^ab^ ± 0.946	16.97 ^abc^ ± 0.293
	Namaqua Afrikaner	36.2 8 ^c^ ± 0.491	14.15 ^a^ ± 0.294	10.39 ± 0.296	36.38 ^b^ ± 0.974	17.60 ^a^ ± 0.302
	South African Mutton Merino	38.85 ^a^ ± 0.541	12.59 ^b^ ± 0.324	10.95 ± 0.326	40.96 ^a^ ± 1.072	16.73 ^bc^ ± 0.333
	*p*-value	<0.001	<0.001	0.391	<0.001	0.027
S × B interaction	*p*-value	0.670	0.439	0.690	0.392	0.727

^a–c^ Column means with different superscripts differ significantly (*p* ≤ 0.05). L*—lightness parameter; a*—redness values; b*—yellowness parameter

## References

[1] Vermeulen H., Schönfeldt H.C., Pretorius B. (2015). A consumer perspective of the South African red meat classification system. S. Afr. J. Anim. Sci..

[2] Department of Africulture, Forestry and Fisheries (2006). Regulations Regarding the Classification and Marketing of Meat in the Republic of South Africa.

[3] Bruwer G.G., Naude R.T., Du Toit M.M., Cloete A., Vosloo W.A. (1987). An evaluation of the lamb and mutton carcase grading system in the Republic of South Africa. 2. The use of fat measurements as predictors of carcase composition. S. Afr. J. Anim. Sci..

[4] Bruwer G.G., Naude R.T., Vosloo W.A. (1987). An evaluation of the lamb and mutton carcase grading system in the Republic of South Africa. 1. A survey of carcase characteristics of the different grades. S. Afr. J. Anim. Sci..

[5] Webb E.C. (2015). Description of carcass classification goals and the current situation in South Africa. S. Afr. J. Anim. Sci..

[6] Red Meat Producers Organisation. http://www.rpo.co.za/information-centre/absa/weekly-prices/.

[7] Pannier L., Gardner G.E., Pethick D.W. (2019). Effect of Merino sheep age on consumer sensory scores, carcass and instrumental meat quality measurements. Anim. Prod. Sci..

[8] Davel M., Bosman M.J.C., Webb E.C. (2003). Effect of electrical stimulation of carcasses from Dorper sheep with two permanent incisors on the consumer acceptance of mutton. S. Afr. J. Anim. Sci..

[9] Brand T.S., Van Der Westhuizen E.J., Van Der Merwe D.A., Hoffman L.C. (2018). Analysis of carcass characteristics and fat deposition of Merino, South African Mutton Merino and Dorper lambs housed in a feedlot. S. Afr. J. Anim. Sci..

[10] Houghton P.L., Turlington L.M. (1992). Application of ultrasound for feeding and finishing animals: A review. J. Anim. Sci..

[11] Erasmus L.J., Botha P.M., Cruywagen C.W., Meissner H.H. (1994). Amino acid profile and intestinal digestibility in dairy cows of rumen-undegradable protein from various feedstuffs. J. Dairy Sci..

[12] Van der Merwe D.A., Brand T.S., Hoffman L.C. Modelling subcutaneous fat deposition in growing South African lambs. Proceedings of the 65th International Congress of Meat Science and Technology.

[13] Cloete J.J.E., Hoffman L.C., Cloete S.W.P., Fourie J.E. (2004). A comparison between the body composition, carcass characteristics and retail cuts of South African Mutton Merino and Dormer sheep. S. Afr. J. Anim. Sci..

[14] Hankins O.G., Howe P.E. (1946). Estimation of Beef Carcass and Cuts. No. 926.

[15] AMSA (2012). Meat Color Measurement Guidelines.

[16] SAS Institute (2006). SAS/Stat User’s Guide Version 9.

[17] Negussie E., Rottmann O.J., Pirchner F., Rege J.E.O. (2003). Patterns of growth and partitioning of fat depots in tropical fat-tailed Menz and Horro sheep breeds. Meat Sci..

[18] Qwabe S.O., van Marle-Köster E., Visser C. (2013). Genetic diversity and population structure of the endangered Namaqua Afrikaner sheep. Trop. Anim. Health Prod..

[19] Owens F.N., Dubeski P., Hanson C.F. (1993). Factors that alter the growth and development of ruminants. J. Anim. Sci..

[20] Cloete J.J.E., Hoffman L.C., Cloete S.W.P. (2012). A comparison between slaughter traits and meat quality of various sheep breeds: Wool, dual-purpose and mutton. Meat Sci..

[21] Kempster A.J. (1981). Fat partition and distribution in the carcasses of cattle, sheep and pigs: A review. Meat Sci..

[22] Butterfield R.M. (1988). New Concept of Sheep Growth.

[23] Dimsoski P., Tosh J.J., Clay J.C., Irvin K.M. (1999). Influence of management system on litter size, lamb growth, and carcass characteristics in sheep. J. Anim. Sci..

[24] Jones M., Hoffman L.C., Muller M. (2015). Oxidative stability of blesbok, springbok and fallow deer droëwors with added rooibos extract. S. Afr. J. Sci..

[25] Kridli R.T., Shaker M.M., Abdullah A.Y., Muwalla M.M. (2007). Sexual behaviour of yearling Awassi, Charollais × Awassi and Romanov × Awassi rams exposed to oestrous Awassi ewes. Trop. Anim. Health Prod..

[26] Burger A., Hoffman L.C., Cloete J.J.E., Muller M., Cloete S.W.P. (2013). Carcass composition of Namaqua Afrikaner, Dorper and SA Mutton Merino ram lambs reared under extensive conditions. S. Afr. J. Anim. Sci..

[27] Khliji S., Van de Ven R., Lamb T.A., Lanza M., Hopkins D.L. (2010). Relationship between consumer ranking of lamb colour and objective measures of colour. Meat Sci..

[28] Hoffman L.C., Muller M., Cloete S.W.P., Schmidt D. (2003). Comparison of six crossbred lamb types: Sensory, physical and nutritional meat quality characteristics. Meat Sci..

[29] Destefanis G., Brugiapaglia A., Barge M.T., Dal Molin E. (2008). Relationship between beef consumer tenderness perception and Warner–Bratzler shear force. Meat Sci..

[30] Webb E.C., O’neill H.A. (2008). The animal fat paradox and meat quality. Meat Sci..

[31] Strydom P.E., Van Heerden S.M., Van Heerden H.C., Kruger R., Smith M.F. (2009). The influence of fat score and fat trimming on primal cut composition of South African lamb. S. Afr. J. Anim. Sci..

[32] Tshabalala P.A., Strydom P.E., Webb E.C., De Kock H.L. (2003). Meat quality of designated South African indigenous goat and sheep breeds. Meat Sci..

[33] Van der Merwe D.A., Brand T.S., Hoffman L.C. (2019). Application of growth models to different sheep breed types in South Africa. Small Rumin. Res..

